# Supplementary material for: A Large Sample Retrospective Study on the Distinction of Voriconazole Concentration in Asian Patients from Different Clinical Departments
Source: Pharmaceuticals (Basel). 2021 Nov 29;14(12):1239. doi: 10.3390/ph14121239 (PMC8705093; doi:10.3390/ph14121239)
Supplement: Supplementary file 1 [file pharmaceuticals-14-01239-s001.zip › pharmaceuticals-1432579 suppl-1126.pdf]

Table S1. Bootstrap method to verify the multiple linear regression model.

Variable	Coefficient (Bootstrap)	Std.Error	Bootstrap P value	95% Confidence Interval		Coefficient (Model)
				Lower	Upper	
Age	0.077	0.022	0.003	0.037	0.123	0.049
ALT	-0.003	0.006	0.554	-0.016	0.010	0.007
TBIL	0.012	0.004	0.002	0.006	0.021	0.010
ALB	-0.034	0.046	0.456	-0.125	0.058	-0.100
GGT	-0.004	0.001	0.010	-0.007	-0.002	-0.004
Constant value	1.827	2.433	0.449	-3.242	6.418	5.195

Dependent variable: voriconazole trough concentration;
Based on 1000 Bootstrap samples

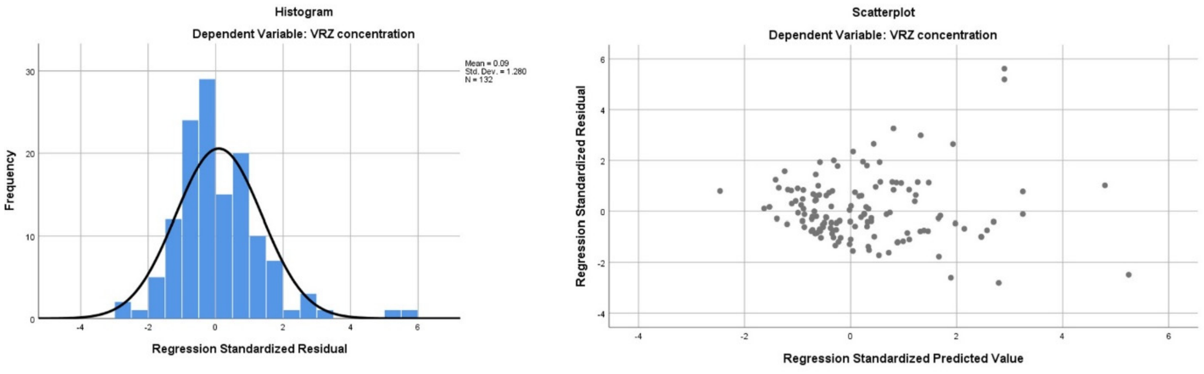

(A) (B)

Figure S1. Model fitting test diagram. Histogram of residual distribution(**A**) and Scatter diagram of residual distribution(**B**)
